# Supplementary material for: A deep learning framework based on structured space model for detecting small objects in complex underwater environments
Source: Commun Eng. 2025 Feb 17;4:24. doi: 10.1038/s44172-025-00367-9 (PMC11833135; doi:10.1038/s44172-025-00367-9)
Supplement: Supplementary file 2 — Supplementary Information [file 44172_2025_367_MOESM2_ESM.pdf]

Supplementary information for

A deep learning framework based on Structured Space Model for detecting small objects in complex underwater environments

Yaoming Zhuang[a,*], Jiaming Liu[a], Haoyang Zhao[a,b], Longyu Ma[a,b], Zirui Fang[c] Li Li[d], Chengdong Wu[a], Wei Cui[e], Zhanlin Liu[f]

[a]Faculty of Robot Science and Engineering, Northeastern University, Shenyang 110819, China
[b]College of Information Science and Engineering, Northeastern University, Shenyang 110819, China
[c]School of Information and Artificial Intelligence, Anhui Agricultural University, Hefei 230036, China
[d]JangHo School of Architecture, Northeastern University, Shenyang 110819, China
[e]Institute for Infocomm Research, Astar 138632, Singapore
[f]AstrumU, Bellevue, Washington, 98004, USA;
E-mail: **zhuangyaoming@mail.neu.edu.cn**

This file includes:

Supplementary Tables 1 to 10.

Test A								
Model	Precision	Recall	holothurian	echinus	scallop	starfish	Map50	Map50-95
UWNet-CBAM	0.801	0.652	0.71	0.87	0.611	0.761	0.738	0.416
UWNet-ECA	0.788	0.659	0.695	0.872	0.62	0.762	0.737	0.417
UWNet-SE	0.778	0.66	0.699	0.863	0.619	0.763	0.736	0.415
UWNet-CA	0.807	0.654	0.719	0.868	0.616	0.764	0.742	0.421
UWNet-EMA	0.778	0.656	0.706	0.871	0.618	0.759	0.739	0.419
UWNet-MSFF	0.819	0.655	0.713	0.875	0.634	0.78	0.751	0.424
Test B								
Model	Precision	Recall	holothurian	echinus	scallop	starfish	Map50	Map50-95
UWNet-CBAM	0.763	0.62	0.599	0.868	0.546	0.757	0.693	0.388
UWNet-ECA	0.772	0.63	0.608	0.865	0.55	0.758	0.695	0.389
UWNet-SE	0.771	0.627	0.621	0.866	0.551	0.756	0.699	0.389
UWNet-CA	0.78	0.629	0.629	0.868	0.563	0.761	0.705	0.395
UWNet-EMA	0.764	0.632	0.622	0.868	0.555	0.745	0.697	0.395
UWNet-MSFF	0.768	0.64	0.637	0.875	0.567	0.76	0.709	0.397

Supplementary Table 1 Impact of replacing MSFF with different attention mechanisms on detection model accuracy

This table presents a comparative analysis of the model's performance when different attention mechanisms (e.g., CBAM, SE, ECA, CA, and EMA) are used as replacements for the proposed MSFF (Multi-Scale Feature Fusion) module (To ensure the fairness of the experimental comparison, the attention mechanisms in the comparative experiments were placed in the same position as the MSFF module.). The results are evaluated on two test sets (Test A and Test B), with performance metrics including Precision, Recall, and the detection accuracy for four target categories (Holothurian, Echinus, Scallop, and Starfish). The data indicate that the model incorporating the MSFF module outperforms the alternatives across most metrics, particularly in terms of overall performance indicators like Map50 and Map50-95, thereby demonstrating the superiority of the proposed MSFF method. (To avoid the influence of different training strategies on the experimental results, we used a unified set of default hyperparameters when comparing the performance of the MSFF module with attention mechanisms)

Datasets		Models	Precision	Recall	mAP50	mAP50-95
URPC2020	TEST-A	YOLOv5s	0.83	0.646	0.715	0.414
		YOLOv7	0.77	0.67	0.735	0.39
		YOLOv8s	0.824	0.638	0.73	0.408
		YOLOv9t	0.793	0.637	0.716	0.402
		YOLOv10s	0.805	0.657	0.742	0.405
		YOLOv11s	0.814	0.663	0.75	0.42
		Mamba-YOLO-B	0.811	0.654	0.739	0.415
		RT-DETR(Res50)	0.791	0.667	0.745	0.413
		baseline	0.789	0.627	0.702	0.392
		Ours	0.793	0.681	0.773	0.44
	TEST-B	YOLOv5s	0.816	0.618	0.675	0.387
		YOLOv7	0.761	0.629	0.69	0.366
		YOLOv8s	0.796	0.61	0.693	0.382
		YOLOv9t	0.782	0.584	0.67	0.371
		YOLOv10s	0.752	0.639	0.694	0.38
		YOLOv11s	0.779	0.634	0.709	0.395
		Mamba-YOLO-B	0.76	0.642	0.689	0.381
		RT-DETR(Res50)	0.779	0.651	0.719	0.396
		baseline	0.782	0.584	0.66	0.362
		Ours	0.777	0.647	0.732	0.413

Supplementary Table 2 Accuracy comparison of different models on two different test sets of URPC2020

Datasets	Models	Precision	Recall	holothurian	echinus	scallop	starfish	mAP50	mAP50-95
DUO	YOLOv5s	0.857	0.78	0.857	0.924	0.667	0.922	0.843	0.622
	YOLOv7	0.863	0.764	0.875	0.929	0.672	0.928	0.851	0.629
	YOLOv8s	0.858	0.766	0.871	0.924	0.683	0.928	0.851	0.676
	YOLOv9t	0.846	0.747	0.846	0.926	0.642	0.926	0.835	0.65
	YOLOv10s	0.883	0.762	0.882	0.928	0.684	0.934	0.857	0.669
	YOLOv11s	0.841	0.788	0.887	0.928	0.689	0.939	0.861	0.686
	Mamba-YOLO-B	0.885	0.75	0.873	0.927	0.674	0.935	0.852	0.67
	RT-DETR(Res50)	0.858	0.733	0.862	0.903	0.635	0.91	0.828	0.609
	baseline	0.841	0.747	0.836	0.922	0.642	0.925	0.831	0.635
	Ours	0.852	0.789	0.893	0.935	0.71	0.944	0.871	0.695
URPC2021	YOLOv5s	0.842	0.757	0.72	0.907	0.795	0.872	0.824	0.473
	YOLOv7	0.833	0.74	0.734	0.912	0.767	0.885	0.824	0.461
	YOLOv8s	0.829	0.767	0.745	0.909	0.813	0.893	0.84	0.507
	YOLOv9t	0.821	0.76	0.731	0.908	0.796	0.889	0.831	0.491
	YOLOv10s	0.834	0.766	0.754	0.912	0.813	0.896	0.843	0.504
	YOLOv11s	0.845	0.769	0.765	0.917	0.817	0.899	0.849	0.515
	Mamba-YOLO-B	0.846	0.769	0.751	0.915	0.816	0.896	0.845	0.515
	RT-DETR(Res50)	0.823	0.733	0.752	0.902	0.811	0.882	0.836	0.485
	baseline	0.828	0.742	0.722	0.903	0.787	0.884	0.824	0.485
	Ours	0.84	0.777	0.773	0.915	0.829	0.904	0.855	0.524

Supplementary Table 3 Accuracy comparison of different models on the URPC2021 and DUO test sets

Datasets	Models	Precision	Recall	mAP50	mAP50-95	Para	GFLOPs	Para(M)
URPC2020	YOLOv5s	0.855	0.775	0.825	0.496	703w	16.5	14.1
	YOLOv7	0.822	0.754	0.822	0.443	933w	26.0	18.1
	YOLOv8s	0.846	0.771	0.846	0.501	1113w	28.4	21.5
	YOLOv9t	0.831	0.759	0.831	0.485	280w	11.7	23.4
	YOLOv10s	0.822	0.783	0.845	0.496	804w	24.5	15.8
	YOLOv11s	0.836	0.773	0.849	0.508	941w	21.3	18.3
	Mamba-YOLO-B	0.823	0.794	0.846	0.501	2180w	49.6	41.9
	RT-DETR(Res50)	0.821	0.784	0.842	0.482	3266w	85.1	65.9
	baseline	0.815	0.764	0.827	0.483	300w	8.1	6.1
	Ours	0.843	0.786	0.865	0.519	667w	21.1	13.5

Supplementary Table 4 Performance comparison of different models on the URPC2020 validation set

Datasets	Models	mAP50				mAP50-95			
		holothurian	echinus	scallop	starfish	holothurian	echinus	scallop	starfish
TEST-A	YOLOv5s	0.695	0.855	0.585	0.726	0.413	0.473	0.343	0.426
	YOLOv7	0.703	0.86	0.615	0.762	0.381	0.434	0.322	0.422
	YOLOv8s	0.69	0.862	0.614	0.754	0.401	0.459	0.348	0.423
	YOLOv9t	0.676	0.847	0.594	0.746	0.399	0.453	0.332	0.422
	YOLOv10s	0.721	0.876	0.615	0.756	0.388	0.468	0.345	0.419
	YOLOv11s	0.737	0.875	0.616	0.772	0.423	0.471	0.35	0.438
	Mamba-YOLO-B	0.731	0.869	0.606	0.75	0.415	0.471	0.341	0.435
	RT-DETR	0.702	0.854	0.624	0.798	0.396	0.45	0.349	0.455
	baseline	0.658	0.848	0.569	0.732	0.382	0.449	0.318	0.42
	Ours	0.741	0.886	0.665	0.799	0.429	0.481	0.384	0.467
TEST-B	YOLOv5s	0.637	0.836	0.518	0.71	0.37	0.457	0.303	0.42
	YOLOv7	0.618	0.868	0.54	0.733	0.335	0.44	0.286	0.404
	YOLOv8s	0.603	0.861	0.558	0.748	0.338	0.463	0.313	0.414
	YOLOv9t	0.568	0.863	0.521	0.729	0.323	0.459	0.291	0.414
	YOLOv10s	0.623	0.864	0.552	0.737	0.331	0.463	0.311	0.415
	YOLOv11s	0.651	0.871	0.55	0.763	0.359	0.474	0.311	0.435
	Mamba-YOLO-B	0.612	0.864	0.545	0.734	0.31	0.463	0.296	0.416
	RT-DETR	0.648	0.865	0.571	0.792	0.36	0.453	0.313	0.456
	baseline	0.563	0.847	0.504	0.726	0.308	0.451	0.28	0.408
	Ours	0.655	0.881	0.609	0.781	0.372	0.474	0.35	0.456

Supplementary Table 5 Detection accuracy comparison across different categories on two test sets

Datasets	Class	Small	Medium	Large
URPC2020 (Test A)	holothurian	142	204	27
	echinus	1709	324	15
	scallop	3001	233	3
	starfish	760	159	4
URPC2020 (Test B)	holothurian	206	240	15
	echinus	3571	638	24
	scallop	3021	170	5
	starfish	1269	220	11
DUO	holothurian	2512	5065	310
	echinus	40168	9485	503
	scallop	984	853	87
	starfish	10122	4341	85
URPC2021	holothurian	2814	3759	266
	echinus	19117	6724	380
	scallop	8988	1560	83
	starfish	7570	2874	103
URPC2020	holothurian	2016	3297	224
	echinus	16119	5928	296
	scallop	5377	1268	75
	starfish	4600	2189	52

Supplementary Table 6 Distribution and quantity of target annotation boxes for various categories across different datasets

Test A								
Model	SODH	SPDConv	MSDBlock	MSFF	Precision	Recall	Map50	Map50-95
Baseline	×	×	×	×	0.789	0.627	0.702	0.392
Baseline-A	√	×	×	×	0.789	0.632	0.715	0.402
Baseline-B	√	√	×	×	0.786	0.639	0.722	0.405
Baseline-C	√	√	√	×	0.809	0.643	0.735	0.414
UWNet	√	√	√	√	0.819	0.655	0.751	0.424
Hyperparameter optimization	√	√	√	√	0.8	0.675	0.773	0.44
Test B								
Model	SODH	SPDConv	MSDBlock	MSFF	Precision	Recall	Map50	Map50-95
Baseline	×	×	×	×	0.782	0.584	0.66	0.362
Baseline-A	√	×	×	×	0.76	0.604	0.669	0.371
Baseline-B	√	√	×	×	0.774	0.61	0.684	0.381
Baseline-C	√	√	√	×	0.762	0.642	0.702	0.392
UWNet	√	√	√	√	0.768	0.64	0.709	0.397
Hyperparameter optimization	√	√	√	√	0.778	0.645	0.732	0.413

Supplementary Table 7 Ablation study results of the model on the two test sets of URPC2020

Model	Para	GFLOPs	Training time	Inference time
UWNet	667w	21.1	160s	10s
Remove MSFF	648w	20.7	150s	10s
Remove MSDBlock	492w	17.3	53s	6s
Remove only HFIB	624w	20.2	149s	9s
Remove only SS2D	563w	19.2	67s	6s

Supplementary Table 8 Evaluation of the Impact of different modules on model performance

Datasets	Models	Precision	Recall	holothurian	echinus	scallop	starfish	mAP50	mAP50-95
URPC2020 (Val)	original	0.842	0.772	0.755	0.916	0.851	0.884	0.851	0.51
	optimization	0.843	0.786	0.788	0.917	0.867	0.888	0.865	0.519
URPC2020 (Test-A)	original	0.819	0.655	0.713	0.875	0.634	0.78	0.751	0.424
	optimization	0.793	0.681	0.741	0.886	0.665	0.799	0.773	0.44
URPC2020 (Test-B)	original	0.768	0.64	0.637	0.875	0.567	0.76	0.709	0.397
	optimization	0.777	0.647	0.609	0.781	0.372	0.474	0.732	0.413
URPC2021	original	0.832	0.766	0.751	0.915	0.82	0.897	0.846	0.512
	optimization	0.84	0.777	0.773	0.915	0.829	0.904	0.855	0.524
DUO	original	0.826	0.805	0.885	0.933	0.692	0.94	0.862	0.68
	optimization	0.852	0.789	0.893	0.935	0.71	0.944	0.871	0.695

Supplementary Table 9 Comparison of model performance before and after hyperparameter optimization

Hyperparameters	URPC2020	URPC2021	DUO
lr0	0.01	0.02	0.02
lrf	0.01	0.01	0.01
momentum	0.937	0.937	0.937
weight_decay	0.0005	0.0005	0.0005
translate	0.1	0.1	0.1
scale	0.5	0.5	0.5
flipud	0.0	0.0	0.0
fliplr	0.5	0.5	0.5
mosaic	1.0	1.0	1.0
mixup	1.0	0.3	0.3
copy_paste	0.0	0.0	0.1
batch_size	8	16	16
close_mosaic	20	10	20

Supplementary Table 10 Hyperparameters used for training the UWNet model on different datasets
